# High-resolution dynamical downscaling experiment outputs data over Reunion and Mauritius islands in the South-West Indian Ocean

**DOI:** 10.1016/j.dib.2023.109665

**Published:** 2023-10-11

**Authors:** Chao Tang, Béatrice Morel, Swati Singh, Alexandre Graillet, Julien Pergaud, Remy Ineza Mugenga, Lwidjy Baraka, Marie-Dominique Leroux, Patrick Jeanty, Mathieu Delsaut, Tyagaraja S.M. Cunden, Girish Kumar Beeharry, Roddy Lollchund

**Affiliations:** a ENERGY-Lab, University of Reunion, 15 Avenue René Cassin CS 92003, 97744 Saint-Denis Cedex 9, Reunion, France; b Laboratoire des Sciences du Climat et de l'Environnement, CEA Paris-Saclay, 91190 Gif-sur-Yvette, France; c Centre de Recherches de Climatologie, UMR6282 Biogéosciences, CNRS/Université de Bourgogne Franche-Comté, 6 boulevard Gabriel, 21000 Dijon, France; d African Institute for Mathematical Sciences Rwanda, KN 3 Rd, Kigali, Rwanda; e Météo-France, Direction Interrégionale pour l'Océan Indien, 97400 Saint-Denis de La Réunion, France; f Department of Electromechanical Engineering and Automation, Université des Mascareignes, Avenue de la Concorde, Rose Hill, Mauritius; g Department of Physics, Faculty of Science, University of Mauritius, 80837 Réduit, Mauritius

**Keywords:** Renewable energy resources, Meteorological information, Downscaling, Regional climate modeling, WRF, Simulation output

## Abstract

The present article describes a dataset encompassing model outputs generated by the Weather Research and Forecasting (WRF) regional climate model. A high-resolution (1km) downscaling simulation was performed over two tropical islands, Reunion and Mauritius, situated in the South-West Indian Ocean (SWIO), with initial and boundary conditions provided by the ERA5 reanalysis with a global resolution of 0.25° × 0.25°. The simulation used three nested domains sequentially configured with spatial resolutions of 9, 3, and 1km, respectively, with a downscaling ratio of 3. The physical configurations of this simulation were determined through previous modeling studies and sensitivity tests. The published simulation data currently covers a period of 10 years, starting from 1991 (with the possibility to be extended to 30 years). Over 60 output variables were selected for publication with open access, including those related to the intermittent energy resources (e.g., surface solar radiation and its direct/diffuse components, wind speed/direction at multiple vertical levels, and precipitation, of interest for the run-off-river hydropower), as well as the widely used climatic/meteorological variables (e.g., temperature, pressure, humidity, etc.) at a temporal resolution varying from a day up to 30 minutes. All the data are available through an open-access data server, where an intelligent algorithm is applied to simplify the download process for data users. For the first time, a long-term, high-resolution climate/meteorological dataset covering Reunion and Mauritius has been simulated and published as open-access data, yielding substantial benefits to studies on climate modeling, weather forecasting, and even those related to climate change in the SWIO region. In particular, this dataset will enable a better understanding of the temporal and spatial characteristics of intermittent climate-related energy resources, consequently facilitating their implementation towards a green and low-carbon future.

Specifications Table

**Table:** 

Subject	Earth and Planetary Sciences
Specific subject area	Climate modeling is a process of using computer models to simulate and predict the behavior of the Earth's climate system.
Type of data	Grid
How the data were acquired	The dataset encompasses model outputs generated by the Weather Research and Forecasting model (WRF, version 4.2.1) of a high-resolution (1km) regional climate simulation covering two tropical islands, Reunion and Mauritius, situated in the South-West Indian Ocean, with initial and boundary conditions provided by the ERA5 reanalysis. The simulation used three nested domains sequentially configured with spatial resolutions of 9, 3, and 1km, respectively. The physical configurations of this simulation were determined through previous modeling and sensitivity studies. The published simulation currently covers a period of 10 years, starting in 1991, with the possibility to be extended to 30 years.
Data format	Analyzed
Description of data collection	The output climate variables are collected directly from the WRF model output.
Data source location	ENERGY-Lab, University of Reunion Saint-Denis, Reunion France
Data accessibility	All data referred to in this data article is publicly available through a THREDDS data server without any access control. Direct URL to data: https://galilee.univ-reunion.fr/thredds/catalog/output/catalog.html DOI: https://doi.org/10.26171/n6bj-w635

## 1 Value of the Data

• This dataset fills the gap in open-access, high-resolution, long-term climate data coverage in Reunion and Mauritius. In addition, given the large number of published climate variables from the model, this dataset has significant potential to advance research in multiple sciences fields, especially in climate-related domains, such as climate change and climate-driven intermittent energy resources. The value of this dataset can be summarized as (but not limited to) the following:
• Weather forecasting: A large regional climate dataset helps in providing detailed information about climate patterns and trends at a regional and even local scale with extended temporal coverage, which is essential for accurate weather forecasting.
• Climate change detection and adaptation: high-resolution climate datasets with long temporal coverage help to identify past climate change and its impacts at local scales, which is crucial for developing adaptation and mitigation strategies, especially for geographically isolated islands.
• Physical process study: high-resolution data allows the analysis of fine-scale processes in climate dynamics, which can reveal new insights into how climate processes work and help to better understand the impacts of climate change at local scales.
• Intermittent climate-related energy resources analysis: long-term and high-resolution data helps to get accurate knowledge of the quantity and spatiotemporal variability of climate-related energy resources, such as solar, wind, and hydro (run-off-river) energy resources, at given locations. That will be valuable for the future production of decarbonized energy with a significant penetration of intermittent energy sources.
• Data validation and evaluation: this dataset with various variables allows for comparison with other datasets, such as ground-based measurements, satellite observations, reanalysis, or outputs from other models, for validation or evaluation purposes.
• Topography impact study: the simulation of two closed, isolated islands with similar climate conditions but contrasting topographies (Fig. 2) allows to study the effects of topography on many aspects of climate, such as vegetation or local wind systems enforced by the topography.

## 2 Objective

The SWIO is a critical maritime zone subject to multiple climatic hazards, such as tropical cyclones, floods, and droughts, which profoundly impact the region's populations and ecosystems [1]. The WRF simulation presented in this article, with a spatial resolution of 1km, can advance climate-related research in the SWIO region by providing the first-ever high-resolution open-access climate dataset. This data can contribute to a better understanding of regional climate variability, extreme weather events [2], and the regional impacts of global climate change [3] and facilitate assessments of renewable energy resources [4] for the energy transition in the region [5,6].

### 2.1 Data description

The dataset consists of selected output variables (see Table 1) from the Weather Research and Forecasting (WRF) regional climate model, restored in the format of Network Common Data Form (netCDF; [7]), which is machine-independent, direct-access and self-describing [8]. Model outputs and corresponding metadata can be easily accessed via programming languages such as Python, R, etc., or graphic user interfaces such as Ncview (http://meteora.ucsd.edu/∼pierce/ncview_home_page.html) or Panoply (https://www.giss.nasa.gov/tools/panoply/). Spatially, the published output variables are either on a single surface level, being in 3 dimensions (time, longitude, latitude), or on the surface and 36 hybrid eta levels in the atmosphere, being in 4 dimensions (time, longitude, latitude, level). These eta levels are calculated by WRF (option AUTO_LEVELS_OPT = 2), with a considerable number of levels spanning the first kilometer of the atmosphere, a region of primary interest for wind energy installation. Temporally, these variables are stored at different frequencies, including 30-minute, hourly, 3-hourly, 6-hourly, and daily. All available variables are listed in Table 1.

**Table 1:** WRF output variables at 1km spatial resolution.

#	Variable name	Unit	Description	Coordinates	Frequency				
1	XLAT	degree_north	latitude, south is negative	XLONG XLAT					
2	XLONG	degree_east	longitude, west is negative	XLONG XLAT					
3	XTIME	minutes	minutes since 1990-01-01 00:00:00						
4	XLAT_U	degree_north	latitude, south is negative	XLONG_U XLAT_U					
5	XLONG_U	degree_east	longitude, west is negative	XLONG_U XLAT_U					
6	XLAT_V	degree_north	latitude, south is negative	XLONG_V XLAT_V					
7	XLONG_V	degree_east	longitude, west is negative	XLONG_V XLAT_V					
8	DZS	m	thicknesses of soil layers				3 h		
9	U	m s-1	x-wind component	XLONG_U XLAT_U XTIME			3 h		30min
10	V	m s-1	y-wind component	XLONG_V XLAT_V XTIME			3 h		30min
11	W	m s-1	z-wind component	XLONG XLAT XTIME			3 h		
12	PH	m2 s-2	perturbation geopotential	XLONG XLAT XTIME			3 h	hour	
13	PHB	m2 s-2	base-state geopotential	XLONG XLAT XTIME			3 h	hour	
14	T	K	perturbation potential temperature theta-t0	XLONG XLAT XTIME			3 h		
15	P	Pa	perturbation pressure	XLONG XLAT XTIME			3 h		
16	PB	Pa	base state pressure	XLONG XLAT XTIME			3 h		
17	Q2	kg kg-1	qv at 2 m	XLONG XLAT XTIME				hour	
18	T2	K	temp at 2 m	XLONG XLAT XTIME				hour	
19	PSFC	Pa	sfc pressure	XLONG XLAT XTIME				hour	
20	U10	m s-1	u at 10 m	XLONG XLAT XTIME	day			hour	30min
21	V10	m s-1	v at 10 m	XLONG XLAT XTIME	day			hour	30min
22	QVAPOR	kg kg-1	water vapor mixing ratio	XLONG XLAT XTIME			3 h	hour	
23	QCLOUD	kg kg-1	cloud water mixing ratio	XLONG XLAT XTIME				hour	
24	QRAIN	kg kg-1	rain water mixing ratio	XLONG XLAT XTIME				hour	
25	QICE	kg kg-1	ice mixing ratio	XLONG XLAT XTIME				hour	
26	QSNOW	kg kg-1	snow mixing ratio	XLONG XLAT XTIME				hour	
27	QGRAUP	kg kg-1	graupel mixing ratio	XLONG XLAT XTIME				hour	
28	TSLB	K	soil temperature	XLONG XLAT XTIME			3 h		
29	SMOIS	m3 m-3	soil moisture	XLONG XLAT XTIME			3 h		
30	SH2O	m3 m-3	soil liquid water	XLONG XLAT XTIME			3 h		
31	SFROFF	mm	surface runoff	XLONG XLAT XTIME			3 h		
32	UDROFF	mm	underground runoff	XLONG XLAT XTIME			3 h		
33	SINALPHA		local sine of map rotation	XLONG XLAT XTIME			3 h		30min
34	COSALPHA		local cosine of map rotation	XLONG XLAT XTIME			3 h		30min
35	HGT	m	terrain height	XLONG XLAT XTIME				hour	
36	TSK	K	surface skin temperature	XLONG XLAT XTIME			3 h		
37	RAINC	mm	accumulated total cumulus precipitation	XLONG XLAT XTIME	day			hour	
38	RAINNC	mm	accumulated total grid scale precipitation	XLONG XLAT XTIME				hour	
39	RAINNCV	mm	time-step nonconvective precipitation	XLONG XLAT XTIME				hour	
40	CLDFRA		cloud fraction	XLONG XLAT XTIME				hour	
41	SWDOWN	W m-2	downward short wave flux at ground surface	XLONG XLAT XTIME			3 h	hour	30min
42	SWNORM	W m-3	normal short wave flux at ground surface (slope-dependent)	XLONG XLAT XTIME					30min
43	GLW	W m-2	downward long wave flux at ground surface	XLONG XLAT XTIME			3 h		
44	SWDDIR	W m-2	shortwave surface downward direct irradiance	XLONG XLAT XTIME				hour	30min
45	SWDDIRC	W m-2	clear-sky shortwave surface downward direct irradiance	XLONG XLAT XTIME				hour	
46	SWDDNI	W m-2	shortwave surface downward direct normal irradiance	XLONG XLAT XTIME				hour	30min
47	SWDDNIC	W m-2	clear-sky shortwave surface downward direct normal irradiance	XLONG XLAT XTIME				hour	30min
48	SWDDIF	W m-2	shortwave surface downward diffuse irradiance	XLONG XLAT XTIME				hour	30min
49	ACSWUPT	J m-2	accumulated upwelling shortwave flux at top	XLONG XLAT XTIME				hour	
50	ACSWDNT	J m-2	accumulated downwelling shortwave flux at top	XLONG XLAT XTIME				hour	
51	ACSWUPB	J m-2	accumulated upwelling shortwave flux at bottom	XLONG XLAT XTIME			3 h		30min
52	ACSWDNB	J m-2	accumulated downwelling shortwave flux at bottom	XLONG XLAT XTIME					
53	ACSWDNBC	J m-2	accumulated downwelling clear sky shortwave flux at bottom	XLONG XLAT XTIME					30min
54	ACLWUPT	J m-2	accumulated upwelling longwave flux at top	XLONG XLAT XTIME				hour	
55	ACLWUPB	J m-2	accumulated upwelling longwave flux at bottom	XLONG XLAT XTIME			3 h		
56	ACLWDNB	J m-2	accumulated downwelling longwave flux at bottom	XLONG XLAT XTIME			3 h		
57	PBLH	m	pbl height	XLONG XLAT XTIME				hour	
58	QFX	kg m-2 s-1	upward moisture flux at the surface	XLONG XLAT XTIME				hour	
59	LH	W m-2	latent heat flux at the surface	XLONG XLAT XTIME			3 h		
60	ACHFX	J m-2	accumulated upward heat flux at the surface	XLONG XLAT XTIME			3 h		
61	ACLHF	J m-2	accumulated upward latent heat flux at the surface	XLONG XLAT XTIME			3 h		
62	T2M_MAX	K	max temperature at 2 m	XLONG XLAT XTIME	day				
63	T2M_MIN	K	min temperature at 2 m	XLONG XLAT XTIME	day				
64	SST	K	sea surface temperature	XLONG XLAT XTIME		6 h			

The names of the WRF output files are made up of elements described above, including variable name, spatial resolution, driven model, and frequency, which are separated by underscores ('_') in the file name and appear in the following order:

$$\begin{array}{ccc} & & \mathrm{Vari}\mathrm{able}\mathrm{Name}\_\mathrm{Doma}\mathrm{in}\_\mathrm{reso}\mathrm{luti}\mathrm{on}\_\mathrm{Driv}\mathrm{enMo}\mathrm{del}\_\mathrm{RCMM}\mathrm{odel}-\mathrm{Vers}\mathrm{ionID}\\ & & \_\mathrm{Freq}\mathrm{uency}\_\mathrm{Star}\mathrm{tTime}-\mathrm{EndT}\mathrm{ime}.\mathrm{nc} \end{array}$$

where *VariableName* corresponds to the variable names as in Table 1; *Domain* is “REU-MAU”, and the *resolution* is “1km”, denoting a domain covering Reunion and Mauritius at 1km spatial resolution; *DrivenModel* is the “ECMWF-ERA5-reanalysis”; *RCMModel-VersionID* is “WRF-v421”; *Frequency* can be one of the following: “30min”, “hour”, “3hr”, “6hr”, or “day”; *StartTime* and *EndTime* are in the format of “YYYYMMDDhhmm” in UTC; and the ‘nc’ suffix stands for the NetCDF format. As an example, an output file can be named as follow:

$$\begin{array}{ccc} & & \text{“}T2\_\mathrm{REU}-\mathrm{MAU}\_1\mathrm{km}\_\mathrm{ECMWF}-\mathrm{ERA}5-\mathrm{rean}\mathrm{alys}\mathrm{is}\_\mathrm{WRF}-v421\_\mathrm{hour}\\ & & \_199201010000-199212310000.\mathrm{nc}\text{''}, \end{array}$$

From the example above, one could know this output file consists of data stored in NetCDF (nc) format of the variable of “2-meter air temperature” (T2) over a domain covering Reunion and Mauritius (REU-MAU) at 1km spatial resolution (1km), in a frequency of hour (hour) from 19920101 to 29921231, taken from a simulation by “WRF-v421” driven by “ECMWF-ERA5-reanalysis”.

## 3 Experimental Design, Materials, and Methods

### 3.1 Initial and boundary conditions and nested domains

**Fig. 1:**
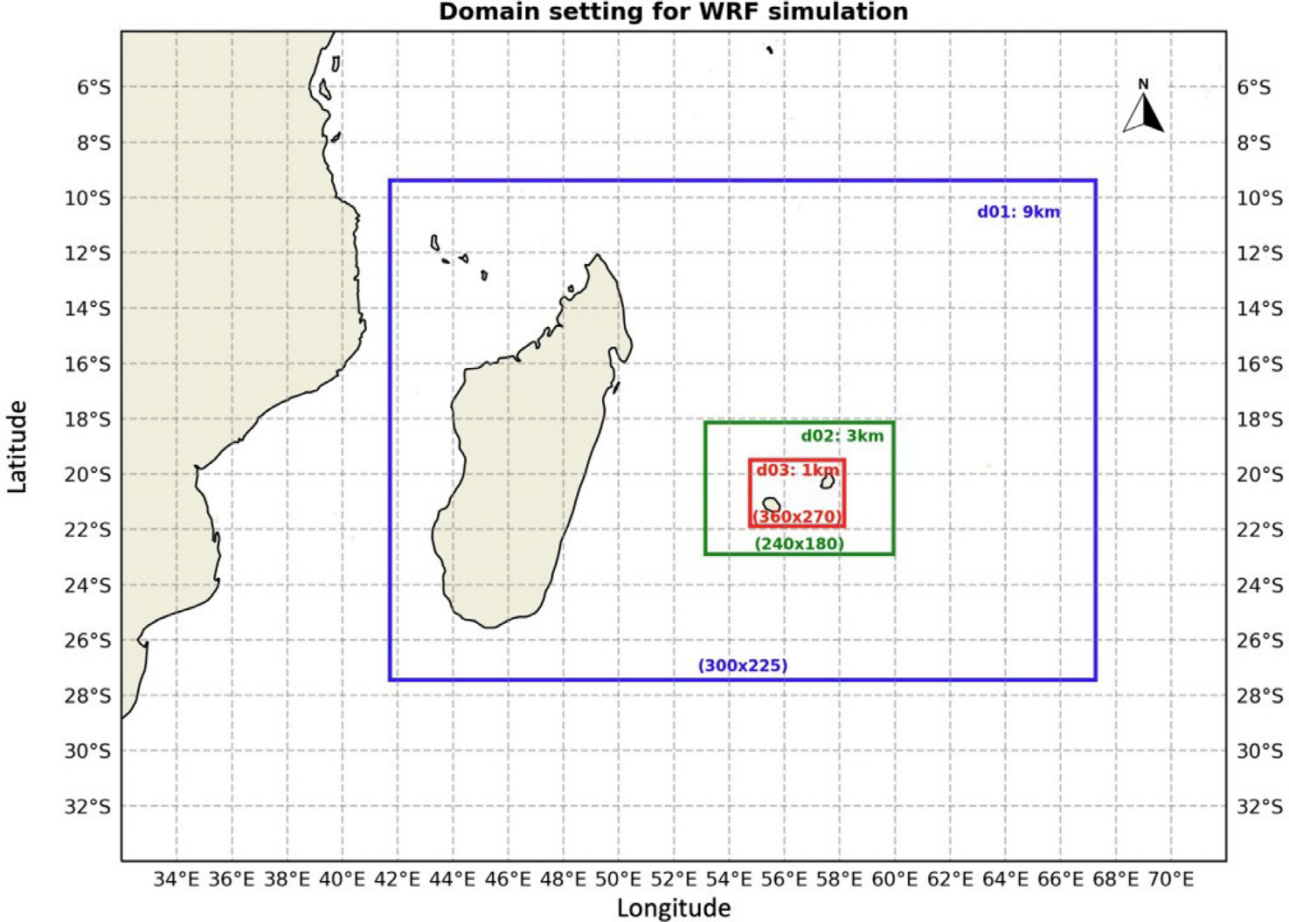
Domain setting of the WRF simulation. Spatial resolution and domain sizes (number of pixels in longitudinal and latitudinal directions) are shown inside each domain.

The non-hydrostatic WRF Model has been used previously in Reunion to represent the general climate conditions [9] and even for extremes [10]. In the present experiment, the dynamical downscaling simulation was conducted with WRF version 4.2.1 [11], driven by 6-hourly ERA5 reanalysis data [12] on pressure levels. Land-use categories were obtained from the Moderate Resolution Imaging Spectroradiometer (MODIS). Three downscaling domains were progressively nested in a one-way mode with increasing horizontal resolutions, as depicted in Fig. 1. The first-guess domain, denoted as d01, which has a mesh size of 300 × 225 pixels in the longitudinal and latitudinal directions respectively, was interpolated by WRF using the ERA5 forcing data. The second domain (d02) has 240 × 181 pixels, and the third one (d03) has 360 × 270 pixels, focusing specifically on the study area, i.e., Reunion and Mauritius.

**Fig. 2:**
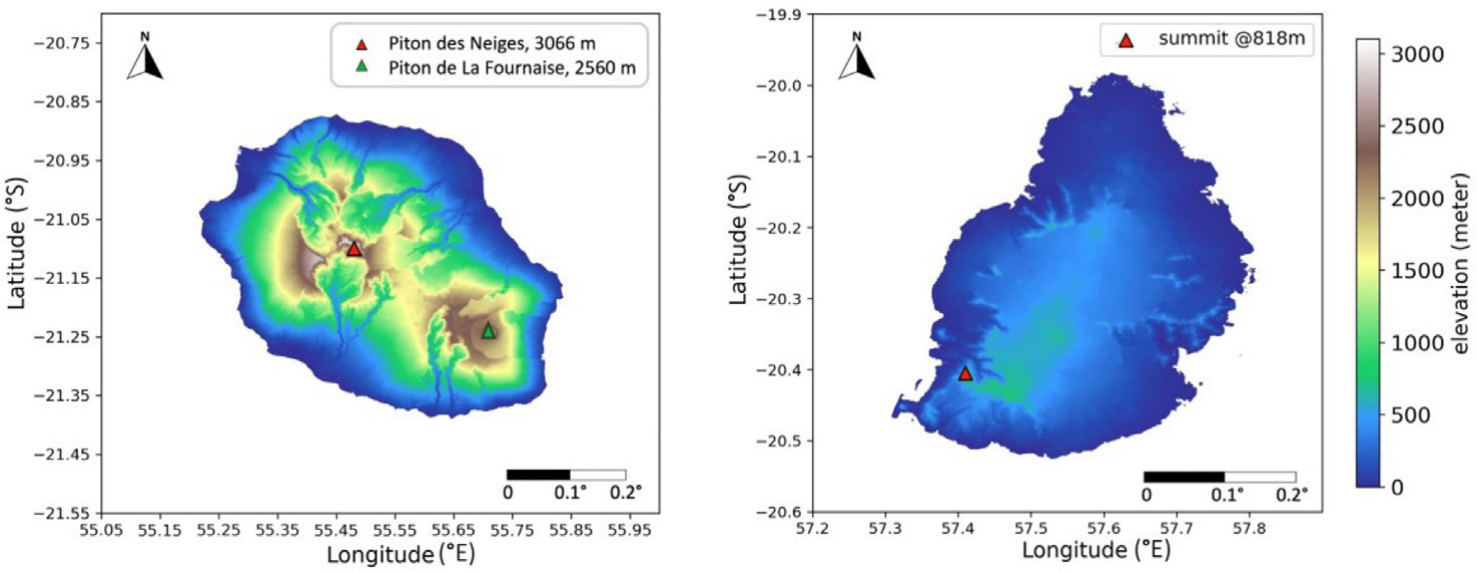
Topography of Reunion (left) and Mauritius (right) in meters based on the ASTER Global Digital Elevation Model from NASA Jet Propulsion Laboratory [13]. The highest summit in Reunion, depicted by a red triangle, is the dormant Piton des Neiges volcano, peaking at over 3000 m asl (above sea level) in the center (red triangle on the left plot). The other one is the active Piton de La Fournaise volcano at 2560 m asl in the east (green triangle on the left plot). Between these two lies a 1500 m-high plateau. On the other side, Mauritius has a relatively flat topography, with a summit measuring about 800 meters in elevation in the southwestern region (indicated on the plot by the red triangle). (For interpretation of the references to color in this figure legend, the reader is referred to the web version of this article.)

### 3.2 Physical configuration

The physical configuration of the WRF simulation has been chosen based on previous simulation studies [9,[14], [15], [16], [17]] and sensitivity tests, in which surface air temperature, surface solar radiation (the energy resource of solar photovoltaic), and 10-meter wind speed and direction were compared with ground-based measurements from the French national meteorological service (i.e., Météo-France) in Reunion and from the Indian Ocean Solar Network (IOS-net, https://galilee.univ-reunion.fr/thredds/catalog.html) in Mauritius. A seasonal comparison based on hourly outputs was performed for 2017, a year without substantial climate variability such as El Niño-Southern Oscillation or Indian Ocean Dipole to avoid the possible large-scale impact in Reunion [18]. The tested physical options are listed in Table 2 (i.e., the column headers), including the Planetary Boundary Layer, Cumulus, Microphysics, and Land Surface Model. The optimal physical configurations of the WRF simulation were then determined based on the results of these sensitivity tests (i.e., the best-performing physical schemes as depicted by a star symbol in each column of that table), along with other physical schemes applied in the simulation but not tested in the sensitivity study, such as the RRTMG scheme for Longwave and Shortwave radiation, and the Monin-Obukhov Similarity scheme for surface-layer option. It is important to note that the cumulus scheme was deliberately deactivated for the second and third domains, where the spatial resolution is fine enough to resolve cloud processes. The configuration file of the WRF model, known as "*namelist.input*", can conveniently be accessed on the identical data server as the model outputs described above in the specifications table.

**Table 2:** Physical schemes tested. The optimal physical schemes are marked by a star (*). Other physical schemes applied in this simulation are not tested in the sensitivity study, such as the RRTMG scheme for Longwave and shortwave radiation and the Monin-Obukhov Similarity scheme for the surface-layer option. The configuration file of the WRF model, specifically named "namelist.input", is conveniently accessible on the identical data server as the model outputs described above in the specifications table.

Planetary boundary layer	Cumulus	Microphysics	Land surface model
Yonsei University scheme (YSU) *	Kain-Fritsch (KF) *	WRF Single-Moment 6-class (WSM6)	Noah
Mellor-Yamada-Janjic scheme (MYJ)	Grell-Devenyi (GD)	Morrison 2-moment (MDM) *	Community Land Model (CLM) *

## Ethics Statements

The presented data did not involve work with humans, animals, or sensitive information.

## CRediT authorship contribution statement

**Chao Tang:** Methodology, Software, Visualization, Formal analysis, Data curation, Writing – original draft, Writing – review & editing, Supervision, Resources, Project administration. **Béatrice Morel:** Conceptualization, Supervision, Resources, Funding acquisition, Writing – review & editing. **Swati Singh:** Methodology, Software, Formal analysis, Validation, Writing – review & editing. **Alexandre Graillet:** Data curation, Writing – original draft, Writing – review & editing. **Julien Pergaud:** Methodology, Software, Resources. **Remy Ineza Mugenga:** Methodology, Software, Data curation. **Lwidjy Baraka:** Data curation. **Marie-Dominique Leroux:** Data curation. **Patrick Jeanty:** Resources, Funding acquisition. **Mathieu Delsaut:** Data curation. **Tyagaraja S.M. Cunden:** Methodology, Software. **Girish Kumar Beeharry:** Methodology, Software. **Roddy Lollchund:** Methodology, Software.

## Data Availability

High-resolution dynamical downscaling experiment outputs data over Reunion and Mauritius islands in the South-West Indian Ocean (Original data) (Galilee).
